# Sufficience serum vitamin D before 20 weeks of pregnancy reduces the risk of gestational diabetes mellitus

**DOI:** 10.1186/s12986-020-00509-0

**Published:** 2020-10-20

**Authors:** Chao-Yan Yue, Chun-Mei Ying

**Affiliations:** Department of Laboratory Medicine, Obstetrics and Gynecology Hospital of Fudan University, Fang Xie Road, No 419, Shanghai, China

**Keywords:** Vitamin D, Gestational diabetes mellitus, Risk factors

## Abstract

**Objective:**

Our aim was to evaluate the relationship between serum vitamin D levels before 20 weeks of pregnancy and the risk of gestational diabetes mellitus.

**Methods:**

This study is a retrospective study. We analyzed the relationship between serum 25 (OH) D level before 20 weeks of pregnancy (first antenatal examination) and the risk of gestational diabetes mellitus. Age, parity and pre-pregnancy body mass index were used as confounding factors. 8468 pregnant women were enrolled in this study between January 2018 and March 2020 at the Obstetrics and Gynecology Hospital of Fudan University. Adjusted smoothing splinespline plots, subgroup analysis and multivariate logistic regression analysis was conducted to estimate the relative risk between 25(OH)D and gestational diabetes mellitus.

**Results:**

After fully adjusting the confounding factors, serum vitamin D is a protective factor in gestational diabetes mellitus (OR = 0.90). Compared with vitamin D deficiency, vitamin D insufficiency (OR = 0.78), sufficience (OR = 0.82) are a protective factor for gestational diabetes mellitus.

**Conclusion:**

Sufficience vitamin D before 20 weeks of pregnancy is a protective factor for gestational diabetes mellitus. Vitamin D > 20 ng/mL can reduce the risk of GDM, which is not much different from the effect of > 30 ng/mL. The protective effect of vitamin D is more significant in obese pregnant women.

## Introduction

Gestational diabetes mellitus (GDM) is a common complication of pregnancy, with an incidence of about 12.9% worldwide [1]. The incidence of gestational diabetes reported in the literature is not consistent in China, with an incidence of 4.3% [2], 9.3% [3], 18.4% [4]. The main physiological functions of vitamin D and its metabolites are to maintain normal blood calcium and phosphorus levels, and ensure bone health and normal neuromuscular function. The extra-skeletal effects of vitamin D include muscle, cardiovascular, metabolism, immunity, tumorigenesis, pregnancy and fetal development [5].Vitamin D synthesizes 25(OH)D in the body catalyzed by 25 hydroxylase, which is the main storage form in the body and reflects the nutritional status of vitamin D in the body. 25(OH)D is hydroxylated at the 1 α position to 25 (OH)_2_D, which is the main active metabolite of vitamin D in the body. It binds to the vitamin D receptor which exists widely in the tissue and plays a hormone-like role. It has been reported that vitamin D plays an important role in pregnancy, such as implantation, immune function, angiogenesis, inflammation and and glucose metabolism. Vitamin D deficiency during pregnancy is widespread worldwide [6–8].

At present, there are few studies on vitamin D deficiency among pregnant women in developing countries, and all the reports of vitamin D deficiency in Chinese pregnant women are after 20 weeks of pregnancy. For example, the vitamin D deficiency rate (< 20 ng/ml) of pregnant women in Wuxi of China is 90% at 23–28 weeks of gestation [9], and the vitamin D deficiency rate (< 20 ng/ml) is 94.7% in Nanjing of China [10], and 90.2% in Beijing [11].

To date, there is no consensus on the exact nature of vitamin D deficiency and gestational diabetes. Although several studies have shown that the lower concentration of 25-hydroxyvitamin D [25 (OH) D] in pregnant women is related to glucose intolerance and the higher prevalence of gestational diabetes [12–18], other studies have not found this contact [19–21]. The prevalence of gestational diabetes in pregnant women with high vitamin D levels is even higher in Guangzhou, China [22]. In addition, the results of existing clinical trials of vitamin D supplementation on gestational diabetes are limited and inconsistent. Two studies have shown that vitamin D supplementation during pregnancy can reduce the incidence of GDM [23, 24], but other studies suggest that vitamin D supplementation has no effect on the incidence of gestational diabetes or maternal blood glucose levels [25–28]. The purpose of our study was to assess the relationship between serum vitamin D and gestational diabetes in Chinese pregnant women before 20 weeks of gestation.

## Methods

### Subjects

A total of 8468 singleton pregnancies women with a live delivery between January 2018 and March 2020 at the Obstetrics and Gynecology Hospital of Fudan University (Shanghai, China) were enrolled in this study. All the pregnant women were from Shanghai (31.23 North latitude). The clinical data and outcomes for mothers and neonates were obtained from clinical records. GDM was diagnosed at 24–28 weeks of gestation using the American Diabetes Association (ADA) criteria [29]. We selected serological examination at the first antenatal visit and age, parity, body mass index (BMI) before pregnancy as confounding factors. Judgment criteria for vitamin D deficiency are as follows: Vitamin D deficiency is defined as a 25(OH)D below 20 ng/mL, and vitamin D insufficiency as a 25(OH)D of 20–30 ng/mL, and ≥ 30 ng/mL is defined as vitamin D sufficience [30]. The study exclusion criteria were as follows: taking calcium and vitamin D supplements before blood collection, diabetes diagnosed before pregnancy, untreated endocrine lesions, stillbirths, or patients without complete maternal and infant records.

### Serum samples

Five milliliters of peripheral blood were collected from fasting participants before weeks 20 of gestation. The peripheral blood was collected in a serum separator tube and samples were allowed to clot for 30 min before centrifugation at 1000 × *g* for 5 min. All peripheral blood samples were processed within 2 h of collection.

### Biochemical analyses

Serum Triglycerides (Tg), total cholesterol (Tch), fasting blood glucose were analyzed by an automatic biochemical analyzer (Hitachi 7180, WAKO) using commercially available kits. Serum 25(OH)D and folate were measured with the Architect i2000 chemiluminescence immunoassay analyzer. Interassay coefficients of variation (CV%) were less than 10% for all these assays. The lower limit of sensitivity for 25-OH-D3 is 3.5 ng/ml.

### Statistical analysis

Data are expressed as mean (SD) for continuous variables and percentage (%) for dichotomous variables. Adjusted smoothing splinespline plots of 25(OH)D by mixed factors were created to study the shape of the relationship of 25(OH)D with the risk of gestational diabetes mellitus. Subgroup analysis examined the relationship between 25(OH)D and the risk of gestational diabetes mellitus according to age and BMI. Test for interaction in the logistic-regression model was used to compare odd ratios between the analyzed subgroups. Logistic-regression models were used to investigate the effects of 25(OH)D and the other variables on the occurrence of gestational diabetes mellitus. The multivariable regression model containing the other variables including age, pre-pregnancy BMI, parity, Tg, Tch. The risk associated with gestational diabetes mellitus is reported according to continuous 25(OH)D per 10 ng/mL and clinical cutoffs of 25(OH)D. Data were analyzed with the use of the statistical packages R (The R Foundation; https://www.r-project.org; version 3.4.3). All *P* values forstatistics were 2-tailed, and a *P* < 0.05 was regarded as statistically significant.

## Results

**Fig. 1:**
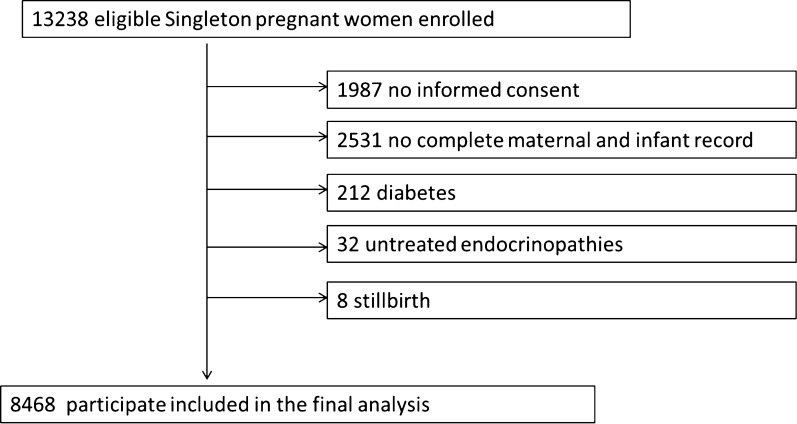
Flowchart of participants

Figure 1 is a flow chart of participant participation. Table 1 describes the baseline characteristics of the subjects, including demographic characteristics and some laboratory test results that may be related to the occurrence of GDM. Among 8468 pregnant women, 48.1% were Vitamin D deficiency, 40% were vitamin D insufficiency, and 11.9% were vitamin D sufficience. The proportion of participants who smoked cigarettes and consumed alcohol was 0%. Additional file 1: Table S1 details the serum vitamin D levels before 20 weeks of pregnancy in different seasons. We found that serum vitamin D in summer and autumn is higher than in spring and winter. Regarding the definition of seasons, we have adopted the division of seasons in the sense of meteorology, based on the value of the daily average temperature reached stably for 5 consecutive days in Shanghai.

**Table 1:** Baseline characteristics of the study participants according to clinical cutoffs concentrations of serum 25(OH)D

Clinical cutoffs	25(OH)D			
	< 20 (ng/mL)	20–30 (ng/mL)	≥ 30 (ng/mL)	*p* value
No. of participants	4070 (48.1%)	3382 (40.0%)	1016 (11.9%)	
GDM, N (%)	492 (12.09%)	329 (9.73%)	98 (9.65%)	
Age (years), mean (SD)	29.17 ± 2.82	29.10 ± 2.81	28.95 ± 2.91	0.099
BMI (kg/m^2^), mean (SD)	21.11 ± 2.87	21.08 ± 2.87	20.74 ± 2.81	< 0.001
Laboratory results mean (SD)				
25(OH)D (ng/mL)	15.10 ± 3.17	24.27 ± 2.75	34.71 ± 4.41	< 0.001
Tg (mmol/L)	1.33 ± 0.56	1.34 ± 0.74	1.32 ± 0.57	0.79
Tch (mmol/L)	4.46 ± 0.73	4.44 ± 0.73	4.39 ± 0.70	0.012
Fasting glucose (mmol/L)	4.51 ± 0.38	4.46 ± 0.35	4.49 ± 0.35	0.015

*BMI* body mass index, *GDM* gestational diabetes mellitus, *Tg* triglycerides, *Tch* total cholesterol

**Fig. 2:**
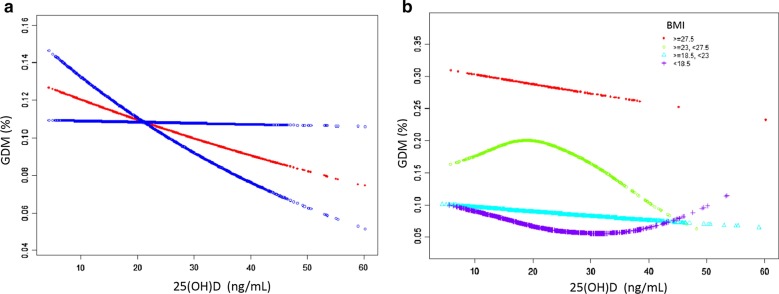
The association between 25(OH)D and GDM. **a** Smooth fitting curve adjusted for age, parity, body mass index, total serum cholesterol, triglycerides, fasting blood glucose. **b** Smooth fitting curve stratified by body mass index, adjusted for age, parity, total serum cholesterol, triglycerides, fasting blood glucose

Figure 2a is a smoothing splinespline plots of 25(OH)D and the risk of gestational diabetes mellitus. Figure 2b is the smoothing splinespline plots after stratification according to pre-pregnancy BMI. Red lines represent the spline plots of Vitamin D concentration and blue lines represent the 95% confidence intervals of the spline plots.

Subgroup analyses are present in Table 2. We found no significant heterogeneity among analyzed subgroups according to age (< 30 or ≥ 30-year old), BMI (< 18.5, 18.5–23, 23–27.5, or ≥ 27.5 kg/m^2^), and season.

**Table 2:** The association between 25(OH)D per 10 ng/mL and GDM according to baseline characteristics

Sub-group	n	OR	95% CI	*p* value	*p* value for interaction
Age					0.80
< 30	5215	0.87	(0.77, 0.98)	0.03	
≥ 30	3253	0.90	(0.77, 1.06)	0.20	
BMI					0.95
< 18.5	1426	0.84	(0.64, 1.10)	0.20	
18.5–23	5270	0.91	(0.80, 1.04)	0.16	
23–27.5	1503	0.90	(0.73, 1.09)	0.27	
≥ 27.5	269	0.84	(0.57, 1.22)	0.36	
Season					0.83
Spring	1641	0.72	(0.51, 1.01)	0.06	
Summer	2748	0.91	(0.81, 1.02)	0.36	
Autumn	1709	0.88	(0.69, 1.12)	0.24	
Winter	2370	0.74	(0.63, 0.87)	< 0.001	

*BMI* body mass index, *PE* preeclampsia, *OR* odds ratio

Table 3 shows the multiple regression results of the effect of vitamin D on gestational diabetes. In the non-adjusted model, every 10 ng/ml increase in serum vitamin D concentration reduced the risk of gestational diabetes by 12%. In the adjust model, every 10 ng/ml increase in serum vitamin D levels reduced the risk of gestational diabetes by 10%. If the concentration of vitamin D is grouped by the clinical cut-off value, compared with vitamin D deficiency, the risk of pregnant women with insufficient vitamin D and sufficient vitamin D decreased by 22% and 18%, respectively, the difference was statistically significant.

**Table 3:** Individual effect of 25(OH)D concentrations on GDM

Exposure	Incidence, n (%)	Non-adjusted		Adjust model	
		OR (95% CI)	*p* value	OR (95% CI)	*p* value
Continuous 25(OH)D per 10 ng/mL	919 (10.85%)	0.88 (0.80, 0.97)	0.009	0.90 (0.81, 0.99)	0.036
Clinical cutoffs					
< 20 (ng/mL)	492 (12.09%)	Reference		Reference	
20–30 (ng/mL)	329 (9.73%)	0.78 (0.68, 0.91)	0.001	0.78 (0.67, 0.90)	0.001
≥ 30 (ng/mL)	98 (9.65%)	0.78 (0.62, 0.98)	0.03	0.82 (0.65, 1.04)	0.102

Adjust model: Adjusted for age, parity, body mass index, total serum cholesterol, triglycerides, fasting blood glucose

## Discussion

Gestational diabetes mellitus is common obstetric complications that endanger maternal and infant health. There is no consensus on the relationship between vitamin D and gestational diabetes mellitus. Our study focused on serum 25(OH)D levels before 20 weeks of pregnancy and evaluated their relationship with Gestational diabetes mellitus. The results showed that serum 25(OH)D before 20 weeks gestation was an independent protective factor for gestational diabetes mellitus in Chinese pregnant women. This is more valuable than studying the relationship between vitamin D concentration and gestational diabetes mellitus after 20 weeks of pregnancy.

Our results are consistent with some existing reports [12–18]. However, a cross-sectional study of the Turkish population found no association between vitamin D deficiency in early pregnancy and the risk of GDM, including 50 GDM cases and 50 controls [19]. Another nested case–control study of 180 pregnant women in North Carolina reported similar findings (60 GDM cases and 50 controls) [20]. These two reports are contrary to our findings, which may be due to the small sample size. A recent review suggests that controversial results of observational studies of vitamin D and gestational diabetes may be affected by the heterogeneity of the study design and inadequate consideration of confounding factors [31].

Our study found that vitamin D deficiency (20–30 ng/mL) and adequate vitamin D (≥ 30 ng/mL) were similar to vitamin D deficiency in reducing the incidence of GDM. This interesting phenomenon is consistent with a recent report that there is a non-linear relationship between the concentration of 25 (OH) D and the risk of gestational diabetes. 50 nmol/L (2.5 ng/mL = 1 ng/mL) may be the threshold concentration of 25 (OH) D, which can determine the correlation between 25 (OH) D concentration and GDM and glucose metabolism. Only when the concentration of 25 (OH) D > 50 nmol/L, they were significantly negatively correlated with GDM risk [32].

In our research results, we also found another interesting phenomenon. The results of smooth fitting curve according to the stratification of pre-pregnancy BMI showed that when the pre-pregnancy BMI was between 23.5 and 27, the risk of GDM changed significantly with the increase of vitamin D, suggesting that clinicians should pay special attention to the concentration of serum vitamin D in obese pregnant women, especially those with high pre-pregnancy BMI. Supplementation of VitD during pregnancy may benefit from reducing the risk of GDM. This phenomenon is similar to that of a recent article. This paper found that maternal VitD deficiency was associated with a higher risk of GDM subtypes with elevated fasting blood glucose, while overweight/obese women were at higher risk [33]. Ou et al. [34] have also reported similar interactions between BMI and VitD and insulin sensitivity, and they observed a stronger correlation between 25 (OH) D and insulin sensitivity in overweight healthy subjects than in normal weight subjects.

In previous studies, the US Institute of Medicine (IOM) guidelines 2009 were used to classify the BMI of women before pregnancy in China [35]. However, World Health Organization experts suggested that the link between body mass index, body composition and health outcomes may be different in Asian and European populations. Studies have shown that for a given BMI, the percentage of body fat in Asians is usually higher than in Europeans. At a relatively low BMI level, the risk of type 2 diabetes, hypertension, and hyperlipidemia in Asian populations has also increased. Based on these observations, it has been suggested that the BMI cut-off point for overweight and obesity in Asian populations should be lower than in European populations. The suggested categories for Asian are as follows: underweight (BMI < 18.5 kg/m^2^); healthy weight (18.5 ≤ BMI < 23 kg/m^2^); overweight (23 ≤ BMI < 27.5 kg/m^2^); obese (BMI ≥ 27.5 kg/m^2^) [29], which is different from IOM guideline. Unlike previous studies, this article focuses on the effects of pre-pregnancy BMI on pregnancy outcomes, classified by Asian criteria.

The pathogenesis of GDM involves many biological processes, the mechanism of vitamin D deficiency in gestational diabetes mellitus is not clear. Vitamin D can influence glucose homeostasis with multiple mechanisms: Functional pancreatic alteration can be associated with immune cell infiltration among glandular cells with consequent inflammation. Vitamin D exerts anti-inflammatory properties that can drive the recovery of physiological insulin secretion. Insulin receptor of peripheral cells drives receptor-mediated endocytosis routing calmodulin-dependent intracellular signalling. Vitamin D enhances duodenal absorption and renal resorption of calcium that is therefore available to the intracellular signalling activated by insulin. Interaction between insulin-like growth facto and molecular partners of vitamin D pathways could play a role in glucose homeostasis. Vitamin D receptors were described in different extra-bones peripheral tissues which explains the wide non-musculoskeletal functions of vitamin D, including the action on insulin receptor that promotes insulin sensitivity. Pancreatic β-cells show VDRs with a possible modulating action of vitamin D on insulin secretion. Vitamin D can act indirectly through the reduction of risk factors common to GDM such as obesity. Vitamin D is a fat-soluble vitamin and its migration from the bloodstream to fat deposits can reduce its availability. Moreover, obese patients have higher levels of vitamin D binding protein that can reduce vitamin D bioactive fractions [28].

Our study found that before 20 weeks of pregnancy, the vitamin D deficiency rate (< 20 ng/ml) of pregnant women in Shanghai was 52.8%, the vitamin D deficiency rate (< 30 ng/ml) was 97.1%, and only 2.9% of pregnant women had adequate vitamin D before 20 weeks of pregnancy. Combined with existing literature reports [9–11], pregnant women in other cities in China, such as Wuxi, Nanjing, and Beijing, have a vitamin D deficiency rate of more than 90% after 20 weeks of pregnancy. This shows that in China, the phenomenon of vitamin D deficiency in pregnant women is very common, and more clinical RCT trials are needed to evaluate the effectiveness and necessity of vitamin D supplementation for pregnant women. Considering that the pathological changes in gestational diabetes mellitus occurred in the earliest pregnancy, the relationship between the serum vitamin levels of pregnant women before pregnancy and the risk of gestational diabetes mellitus needs to be further studied.

Our article has the following shortcomings, The information about the family history and history of gestational diabetes of some pregnant women is missing, which affects the accuracy of the model. Therefore, it is not analyzed as a confusing factor. We did not provide the serum calcium concentration in pregnant women before 20 weeks of pregnancy. This study was conducted in Shanghai so the results cannot extrapolate to other Chinese cities or locations.

## Conclusion

The level of vitamin D in pregnancy is significantly related to the outcome of gestational diabetes mellitus. Vitamin D > 20 ng/mL can reduce the risk of GDM, which is not much different from the effect of > 30 ng/mL. The protective effect of vitamin D is more significant in obese pregnant women. Clinicians should pay more attention to Vitamin D deficiency pregnant women and strengthen the supervision of pregnancy, which has important clinical significance to reduce gestational diabetes mellitus and avoid adverse pregnancy outcome.

## Supplementary information

**Additional file 1.** Serum vitamin D levels in different seasons before 20 weeks of pregnancy.

## Data Availability

The datasets used and/or analysed during the current study are available from the corresponding author on reasonable request.

## Abbreviations

BMI: Body mass index
GDM: Gestational diabetes mellitus
Tg: Triglycerides
Tch: Total cholesterol
CV%: Coefficients of variation
