# Supplementary material for: Sufficience serum vitamin D before 20 weeks of pregnancy reduces the risk of gestational diabetes mellitus
Source: Nutr Metab (Lond). 2020 Oct 20;17:89. doi: 10.1186/s12986-020-00509-0 (PMC7574245; doi:10.1186/s12986-020-00509-0)
Supplement: Supplementary file 1 — Additional file 1. Serum vitamin D levels in different seasons before 20 weeks of pregnancy. [file 12986_2020_509_MOESM1_ESM.docx]

Supplementary Table S1. Serum vitamin D levels in different seasons before 20 weeks of pregnancy

| Season | n | | 25(OH)D (ng/mL) | 2018 | 2019 | 2020 |
| --- | --- | --- | --- | --- | --- | --- |
| spring | | 1641 | 19.62± 6.07 | 2018.3.12-2018.5.11 | 2019.3.9-2019.5.18 | 2020.2.16-2020.5.17 |
| summer | | 2748 | 21.15± 6.47 | 2018.5.12-2018.10.4 | 2019.5.19-2019.10.13 |  |
| autumn | | 1709 | 21.46± 7.25 | 2018.10.5-2018.12.6 | 2019.10.14-2019.12.4 |  |
| winter | | 2370 | 20.79± 8.75 | 2018.1.1-2018.3.11 | 2018.12.6-2019.3.9 | 2019.12.5-2020.2.16 |
